# Sucrose Esters and Beeswax Synergize to Improve the Stability and Viscoelasticity of Water-in-Oil Emulsions

**DOI:** 10.3390/foods12183387

**Published:** 2023-09-09

**Authors:** Mingjun Shu, Yuling Zhou, Yuanfa Liu, Liuping Fan, Jinwei Li

**Affiliations:** State Key Laboratory of Food Science and Technology, Jiangnan University, Wuxi 214122, China; 6210113206@stu.jiangnan.edu.cn (M.S.); zhouyuling@jiangnan.edu.cn (Y.Z.); foodscilyf@163.com (Y.L.)

**Keywords:** oil dispersions, wax, crystals, water-in-oil, rheological characterization, emulsion gels

## Abstract

W/O emulsions are commonly used to prepare stable low-fat products, but their poor stability limits widespread applications. In this study, sucrose ester (SE) and beeswax were utilized to prepare an oil dispersion system in rapeseed oil, which was used as the external oil phase to further synergistically construct the W/O emulsion systems. The results show that spherical and fine crystals are formed under the synergistic effect of SE and BW (1.5 SE:0.5 BW). In this state, a dense interfacial crystal layer was easily formed, preventing droplet aggregation, leading to droplet size reduction (1–2 μm) and tight packing, improving viscoelasticity and resistance to deformation, and increasing the recovery rate (52.26%). The long-term stability of W/O emulsions containing up to 60 wt% water was found to be more than 30 days. The increase in the aqueous phase led to droplet aggregation, which increased the viscosity (from 400 Pa·s to 2500 Pa·s), improved the structural strength of the emulsion, and increased the width of the linear viscoelastic region (from 1% strain to 5% strain). These findings provide some technical support for the further development of stable low-fat products.

## 1. Introduction

Water-in-oil (W/O) emulsions are a class of thermodynamically unstable systems in which droplets of the internal water phase are dispersed in the external oil phase. They are widely used in the cosmetics, pharmaceutical and food industries [1]. They can also encapsulate and deliver nutrients and act as a microreactor for some enzymes [2,3]. They are also used in developing new low-fat foods [4]. However, W/O emulsion systems typically undergo a combination of emulsification/precipitation, aggregation, binding, and Ostwald maturation, resulting in decreased stability, which can be improved by reducing the particle size of the droplets, constructing an oleogel of the outer oil phase, and adding emulsifiers. [5,6]. Therefore, achieving the long-term stability of W/O emulsions is still a difficult task, which also restricts the further development and application of W/O emulsion systems in the food industry [7].

For W/O emulsions, non-ionic surfactants with low hydrophilic and hydrophile-lipophile balance (HLB) are usually used, so spatial stabilization is considered to be the main stabilization mechanism [8]. Some of the unusual emulsifiers, such as glycerin monostearate and sodium stearoyl lactylate, can form crystals in the oil phase and further form oleogels, but the concentrations needed to form oleogels are higher. Although sucrose ester (SE), a food-grade emulsifier, can form crystals in the oil phase, it cannot form a network oleogel, but only a turbid oil phase. In SE, which is made from pure sugar and vegetable oil, the sucrose segment acts as the hydrophilic group and the fatty acid acts as the lipophilic group [9]. According to the length of the fatty acid chain, sucrose esters can have different HLB values [10]. Compared with other chemically synthesized surfactants, SE has good environmental compatibility, as well as non-toxic, biocompatible and non-allergic properties [11], and is widely used as an emulsifier and stabilizer in food, cosmetics and pharmaceutical industries [12]. Viscoelasticity is related to the particle size of the emulsified droplets. The smaller the droplets, the easier it is for them to stack together, enhancing viscoelasticity, and the better the stability. Xin Hu et al. [13] prepared W/O Pickering emulsions using spherical crystals of C-1801 as particles. Although the prepared emulsion was gel-like, SE alone could not form a network structure, resulting in a loose internal structure of the prepared emulsion, which displayed weak viscoelasticity, was vulnerable to external stress and had poor stability. Therefore, it is necessary to add substances that enhance the structural strength of the emulsion, thereby improving its viscoelasticity and stability.

Waxes are mainly used as a gelling agent to form oleogels. They consist mainly of wax esters, n-alkanes, fatty acids and fatty alcohols, are hydrophobic substances with a high melting temperature and are solid at room temperature [14]. The crystalline nature of waxes contributes to the stability of the oil–water interface to some extent. Although it has many advantages, the construction of stable oleogel and W/O emulsions usually requires a high concentration of waxes. The high content of waxes alters the rheological properties of W/O emulsions, giving them a somewhat waxy and brittle structure and reducing deformation resistance. Vazquez et al. [15] used monoglyceride and candle wax (CLM) as gel factors to prepare oleogel and W/O emulsion gel, and found that monoglyceride reduced the interaction between oil gel and emulsion gels crystals. Teodoro et al. [16] prepared mixed oleogel and emulsion gels with CLW, monoglyceride and stearic acid, and found that a single CLW oleogel could stabilize the water phase containing 20% W/O emulsified gels. Although the samples they prepared showed excellent stability, the concentration of wax used was higher. OgütCü et al. [17] prepared oleogel and emulsion gel from olive oil and BW, and found that the emulsion gel had moderate hardness and viscosity. However, the BW content in the prepared samples was too high at 5%, which may lead to poor taste in later food applications.

The main purpose of this study is to explore the introduction of BW network structure into SE systems, and thereby enhance the internal structural strength of W/O emulsions, and improve their viscoelasticity and stability. The crystal morphology of SE and BW in the oil phase was observed by polarizing microscope. The effects of different conditions (SE concentration, SE/BW ratio, and aqueous phase content) on the prepared W/O emulsion systems were investigated by combining the microstructural characteristics and macroscopic rheological properties of the emulsion systems. On this basis, the interrelationship between the macroscopic properties and the microstructure of the emulsions was elaborated.

## 2. Materials and Methods

### 2.1. Materials

Sucrose ester (S-10, HLB = 1, composed of 1% monoester and 99% di-/tri-poly esters) was purchased from Zhejiang Hetang Technology Co., Ltd. (Jinhua, China). Beeswax (acid value: 20.0 mg KOH/g, iodine value: 10.0 g I_2_/ 100 g, and melting point: 65 °C) was kindly donated by Likangweiye Technology Co., Ltd (Beijing, China). Rapeseed oil was purchased from Yihai Kerry Investment Co., Ltd. (Shanghai, China). Nile Red was obtained from Sigma-Aldrich (St. Louis, MO, USA). W/O emulsion was prepared with distilled water as the internal phase.

### 2.2. Preparation of Oil Dispersions and W/O Emulsions

Five SE to BW ratios (2% SE, 1.5% SE: 0.5% BW, 1% SE: 1% BW, 0.5% SE: 1.5% BW, 2% BW) oil dispersions were prepared. Firstly, the required amounts of SE and BW were accurately weighed and dispersed in the rapeseed oil. The beaker containing the mixture was then heated at a temperature above the melting point of SE and BW (>70 °C) with the help of a multi-point magnetic stirrer (TA Instruments, New Castle, DE, USA) stirring at 500 rpm for more than 30 min to achieve melting. Finally, the homogeneous mixture was placed in ice water to cool. Subsequently, these samples were stored in a 5 °C refrigerator for further experiments.

First, different concentrations of SE and different ratios of SE to BW were dissolved in rapeseed oil at 80 °C. The oil phase was then emulsified with preheated water (>80 °C) for 5 min at 1000 rpm with magnetic stirring. The crude emulsion was then homogenized by stirring with an Ultra-Turrax disperser (IKA, Staufen, Germany) at 11,000 rpm for 3 min. The emulsion samples were prepared with different concentrations of SE, different proportioning conditions and with different water contents (20, 30, 40, 50, 60 wt%). All the emulsion samples were stored at 5 °C for further experiments. They could be stored for more than 30 days.

### 2.3. Microstructure Observation

The microstructure of the oil dispersions was observed in polarized light mode using a Leica microscope (DM 2700 P, Leica, Germany) which was fitted with a charge-coupled device camera (DFC 450, Leica, Germany). The samples were heated and melted at 80 °C to eliminate crystal memory, and then a drop of the freshly melted sample was added to a preheated microscope slide and covered with a coverslip. The microscope samples were then transferred to a 5 °C refrigerator and held for 24 h prior to testing.

The microstructure of the emulsion was captured by a DM 2700 P Leica microscope in bright field mode and then observed in the same field in dark field mode to clarify the crystal position. To measure the droplet size of the W/O emulsions, at least 300 droplets were randomly selected in the optical microscope images and analyzed by Image J to obtain droplet size distribution curves and average droplet sizes [18]. The microstructure of emulsion droplets with different percentages of water phase was photographed by inverted fluorescence microscope (Axio Vert A 1, Zeiss, Germany).

For confocal laser scanning microscopy (CLSM), a fluorescent dye solution was prepared by dissolving Nile red in ethanol (1 mg/mL). Then the Nile red solution was added to the emulsion to stain the oil phase. A Zeiss LSM 710 confocal laser scanning microscope (Carl Zeiss Inc., Oberkochen, Germany) was used to take pictures to observe the microstructure, and a 514 nm argon laser was used to excite Nile Red, which is marked in the images as red.

### 2.4. X-ray Diffraction (XRD) Analysis

A Bruker D2 Phaser X-ray diffractometer (AXS Inc. Karlsruhe, Germany) equipped with Cu-Kα radiation (λ = 1.5406 Å) and a Ni filter was used to characterize the polymorphic crystalline forms of the oil dispersions at room temperature. For wide-angle X-ray diffraction analysis (WAXD), the oil dispersion samples were placed in a sample holder and the excess oil dispersion was scraped off before measurement. The samples were scanned in the 2θ angle range from 1 to 35 with a step size of 0.05. The data obtained were analyzed by MDI Jade 6 software (material data Inc., Livermore, CA, USA).

### 2.5. Fourier Transform Infrared Spectroscopy (FTIR) Analysis

The FTIR spectra of oil dispersion, rapeseed oil, SE and BW were collected using a Nicolet IS-10 FTIR spectrometer (Thermo Nicolet, Beijing, China) and an attenuated total reflection (ATR) crystal attachment. The test procedure described in detail by Meng et al. [19] was applied with minor modifications. All the samples were scanned in the wave number range of 4000 cm^−1^ to 650 cm^−1^. The results were then obtained by subtracting the air background spectra and analyzed by OMSNIC (Thermo, v 8.0) software.

### 2.6. Rheological Analysis

The rheological properties of the samples were measured using a rheometer (DHR-3, TA Instruments, Massachusetts, USA) equipped with parallel plates with a diameter of 40 mm and a gap of 1000 μm, and the temperature control unit was the Peltier system. A suitable amount of sample was loaded onto the lower plate of the rheometer and allowed to stand for 30 s before testing to eliminate the effects of external forces. All the samples were analyzed at 25 °C.

For the oil dispersions, the shear rate was increased from 0.1 to 100 s^−1^, and the viscosity was tested by flow scanning. Next, the linear viscoelastic region (LVR) of the oil dispersions was determined by strain scanning in the range of 0.001–100% at a fixed frequency of 1 Hz. The storage modulus (G′) and loss modulus (G″) of the oil dispersions were measured at a rate of 5 °C/min from 5 °C to 80 °C using a strain amplitude of 0.1% and a frequency of 1 Hz.

For the emulsions, the same flow scanning experiments as the oil dispersions were carried out. Next, the LVR of the samples was determined by strain scanning in the range of 0.01~100% at a fixed frequency of 1 Hz. Then, under a constant strain amplitude (0.1%), the changes in the viscoelastic modulus with angular velocity (0.1~100 rad/s) were recorded in the LVR. A three-part thixotropy test (3-itt) was carried at 25 °C, and the viscosity curves were measured at low shear rates (10 or 0.1 s^−1^) of 300 s and 100 s, respectively. The recovery rate was calculated by dividing the viscosity at the end of the third stage by the peak viscosity of the first stage. Then, a creep-recovery experiment was carried out. The emulsions were stressed at 10 Pa for 300 s, and recovery of the samples was then measured for 300 s after the stress was removed. In addition, large amplitude oscillatory shear (LAOS) tests were conducted outside the LVR, and the original stress–strain waveform data were collected at a frequency of 1 Hz.

### 2.7. Statistical Analysis

All trials were repeated three times and the data are presented as mean ± standard deviation. The data analysis and graphing were performed by SPSS and Origin 2021 software, respectively. The significance of differences was determined by Duncan’s multiple comparison test and was considered significant at *p* < 0.05.

## 3. Results and Discussion

### 3.1. Related Characterization of Oil Dispersions

**Figure 1:**
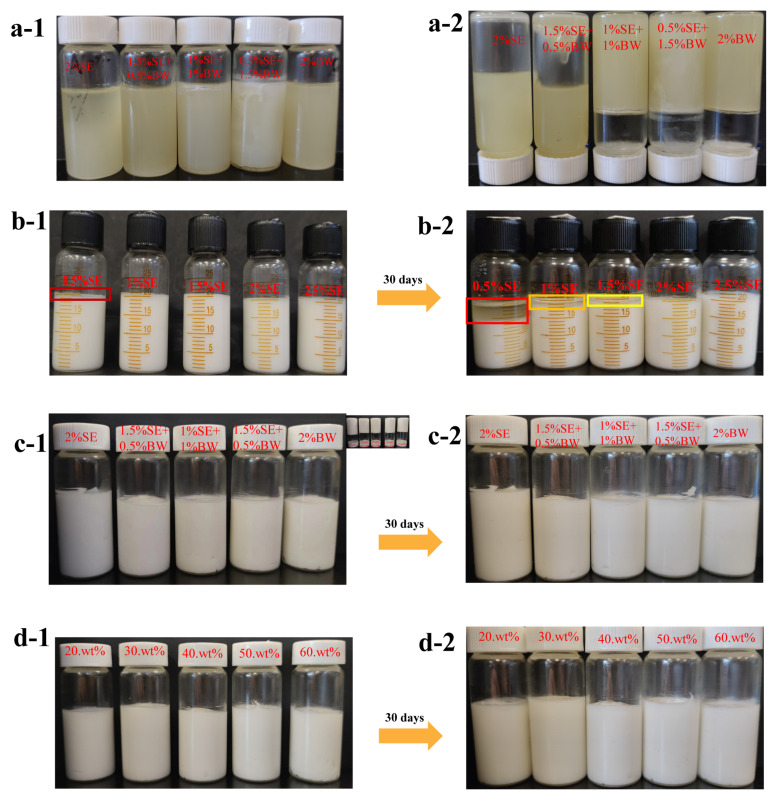
The visual appearance of oil dispersions in different ratio systems of SE and BW (**a-1**,**a-2**); W/O emulsions prepared with SE with different concentration gradients (**b-1**,**b-2**), at different SE and BW ratios (**c-1**,**c-2**), and based on 1.5% SE and 0.5% BW complex systems with different aqueous phases (**d-1**,**d-2**).

**Figure 2:**
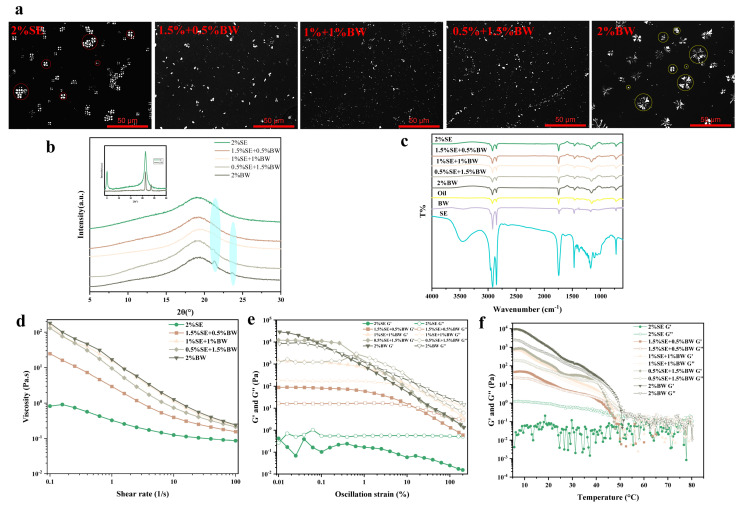
Microscopic images of crystals under PLM (**a**, scale bar = 50 μm); XRD pattern (**b**); FTIR pattern (**c**); and macroscopic rheological pattern (**d**–**f**) of oil dispersions in different ratio systems of SE and BW.

For the oil dispersions with different SE and BW ratios, direct visual evaluation was conducted after storage at 5 °C for 24 h, and the inverted vials filled with samples were used to determine whether the oil dispersions of each system formed a network structure. Figure 1a shows the appearance of the oil dispersions for different SE and BW proportioning systems. It can be seen that the oil dispersion for the single SE system was that of a turbid fluid, indicating that SE could only precipitate crystals in the oil phase and could not form further network structures. The single BW system did not flow even when inverted, implying that a dense network structure was formed within the system, which was an oleogel system (Figure 1a). When BW was introduced into an SE system, it added a certain amount of network structure to the SE system which could not form a network structure. A small amount of BW in the SE system (1.5% SE: 0.5% BW) could form a weak gel network structure. Next, the polarized light microscope (PLM) was used to characterize the microscopic crystal morphology of the oil dispersions in each system. As shown in Figure 2a, the SE and BW crystals showed birefringence, with rapeseed oil as the dark background. In the oil dispersions with a single SE system, the crystal shape of the SE in the oil phase was a spherical crystal (circled in red) with Maltese cross characteristics. Xin Hu et al. [13] dissolved sucrose stearate C-1801 in edible oil and similar spherical crystals were observed. It has been observed that single BW systems in rapeseed oil show the large spherical crystal morphology of BW. Sarbojeet Jana and Silvana Martini [20] found BW to consist of large spherical crystals in canola, soybean and sunflower oils and needle-like crystals in olive oil, corn oil and safflower oil. In the PLM plots of the mixed systems, no distinct spherical crystal patterns were observed for SE and BW, but some larger, similar spherical crystal patterns were observed in the 1.5% SE and 0.5% BW systems. However, in the mixed systems with higher BW content, both SE and BW crystals showed small crystal dispersions in the oil phase. The comparison shows that when the BW content exceeded that of SE, the content of fine crystals in the system was increased and the number of more pronounced agglomerates was reduced, so that the more pronounced spherical crystals could not be revealed, which was the same phenomenon reported by S’anchez-Becerril et al. [21].

In order to obtain further structural information on the crystal network, the oil dispersions of different mixed systems, and the SE and BW powders were analyzed by XRD. As shown in Figure 2b, stronger diffraction peaks were present at 5° and 21.3° for the SE powder and at 21.3° and 23.6° for the BW powder. By comparing the XRD spectra of the oil dispersion, SE powder and BW powder, it was found that the diffraction peaks of the single BW system appeared at the same angle as those of the BW powder, indicating that the BW crystals were entangled together to form a network structure at this time and would not affect the stacking arrangement of the BW crystals. However, in the single SE system, no sharp diffraction peaks were found at the corresponding positions. Only broad peaks (around 20°) were found, and only weak diffraction peaks of BW crystals were found in the system with BW content greater than SE content, which may be due to the lower concentration in the oil phase, the smaller particle size of the crystals, and the lower crystallinity of the crystals. The XRD patterns cannot show the exact crystal structures of both SE and BW, so the polarized light microscope and FTIR spectra were used to obtain further evidence. FTIR analysis was carried out on samples of rapeseed oil, SE powder, BW powder and oil dispersions in different ratios and the spectra are shown in Figure 2c. The smaller peak around 3006 cm^−1^ in the FTIR spectra of canola oil is the characteristic peak of the stretching vibrations of = C-H in canola oil [19]. Transmission peaks around 2920 cm^−1^ and 2850 cm^−1^ were observed in SE powder, BW powder and rapeseed oil, which are associated with C-H stretching vibrations. There was a clear and prominent peak around 1743 cm^−1^, which was characteristic of C=O stretching vibrations, indicating the existence of ester bonds. In the spectrogram of the BW powder, only the C-bending of CH3 at 1463 cm^−1^ was observed, but the C-H bending of CH3 and CH2 at 1463 cm^−1^ and 1377 cm^−1^ were observed in the spectrogram of rapeseed oil and SE powder. The characteristic peak at 1160 cm^−1^ was related to the C-O stretching vibration of C-O-H. In addition, the characteristic peak at 720 cm^−1^ was related to the (CH2) _n_ bending vibration of the alkyl chain. The FTIR spectra showed that the position of the FTIR peak in the wavenumber range 4000–650 cm^−1^ was not affected by the ratio of SE to BW, and that infrared spectra were much the same, both showing characteristic peaks similar to those of rapeseed. A comparison of the IR spectra of SE powder, BW powder and rapeseed oil showed that no new characteristic peaks were formed and no characteristic peaks disappeared for the oil dispersions of the different proportioning systems, suggesting that other weak non-covalent interactions, i.e., van der Waals forces, were mainly at work in the system of oil dispersions, whether mobile or solid-like [18,22].

Figure 2d–f show the results of shear flow scanning, strain scanning and temperature scanning of oil dispersions with different proportions. As shown in Figure 2d, the viscosity of the oil dispersion decreases with increasing shear rate (0.1–100 s^−1^), which is typical shear thinning behavior. This may be due to the fact that some aggregates in the oil dispersion are destroyed at high shear rates, and the crystal particles undergo orientation rearrangement under shear, resulting in viscosity reduction [23,24]. The results of the strain scanning in Figure 2e show that the oil dispersions containing BW were within a certain strain range (i.e., LVR), and had certain gel characteristics. The higher the BW ratio, the higher the G′, but at the same time, the smaller the LVR. Therefore, oil dispersions with suitable LVR and viscoelasticity could only be obtained by adding the right amount of BW (i.e., 1.5% SE: 0.5% BW) to the SE system. This indicates that in the single BW system, the BW crystals were susceptible to damage by external deformation, although they could form network structures and gelled in the oil. The temperature scanning curve is shown in Figure 2f, where the single SE system was in a “liquid state” (G′ ≤ G″). For the BW system, although the modulus decreased with the increase in temperature, G″ was still higher than G′. The intersection of G′ and G″ was considered to be the gel-sol transition temperature. The gel-sol transition temperature of the 1.5% SE and 0.5% BW system was about 37 °C, with good oral melting properties [25].

### 3.2. Related Characterization of Emulsions

#### 3.2.1. Appearance and Microstructure of W/O Emulsions

**Figure 3:**
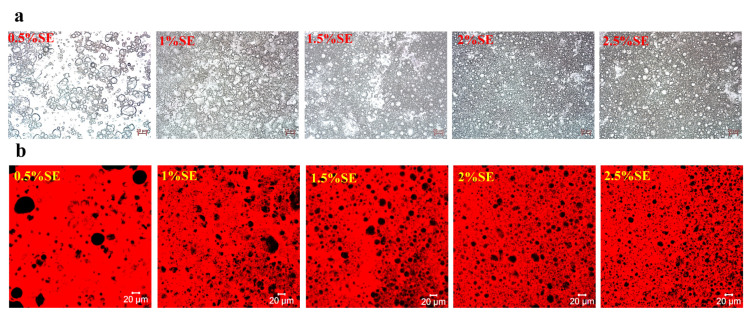
Microscopic images of BM (**a**) and CLSM (**b**) of W/O emulsions with different SE concentration gradients (scale bar = 20 μm).

**Figure 4:**
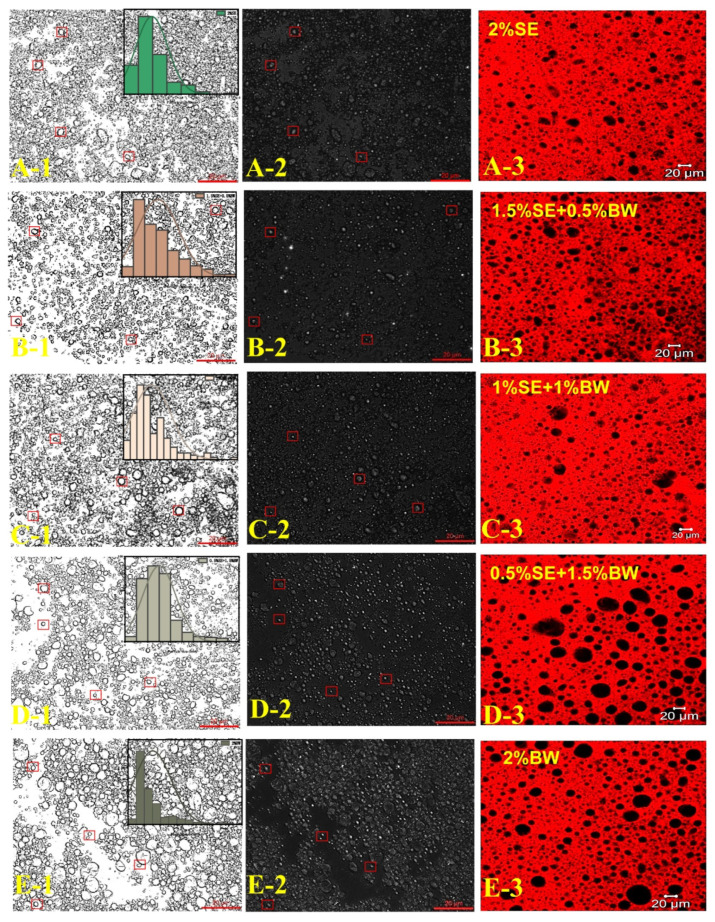
Microscopy images of BM (**A-1**,**B-1**,**C-1**,**D-1**,**E-1**), PLM (**A-2**,**B-2**,**C-2**,**D-2**,**E-2**) and CLSM (**A-3**,**B-3**,**C-3**,**D-3**,**E-3**) of W/O emulsions in different ratio systems of SE and BW (scale bar = 20 μm).

**Figure 5:**
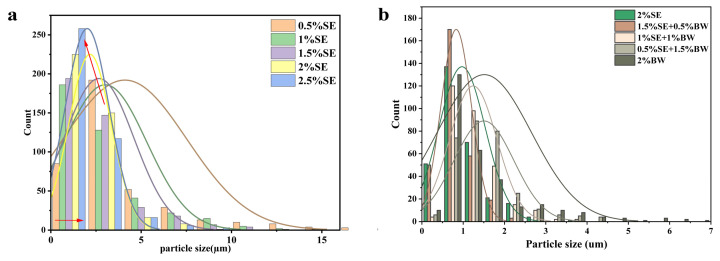
(**a**) The particle size distribution of W/O emulsions with different SE concentrations; (**b**) particle size distribution of emulsions with different proportions of SE and BW.

Firstly, W/O emulsions with 40 wt% of the aqueous phase were prepared using different concentrations of SE. Figure 3 shows the optical microscopy and CLSM images of the W/O emulsions at different SE concentration gradients. Figure 4 shows the appearance and droplet distribution of W/O emulsions in this system. In Figure 1b-1,b-2, it can be seen that only SE concentrations greater than 2% resulted in more stable emulsions, while all emulsions with concentrations below 2% showed varying degrees of oil leakage. Figure 3a and Figure 5a show the optical microscope images and particle size distributions, respectively, of W/O emulsions with different SE concentrations. It can be seen that the emulsion droplets gradually became smaller and the particle size decreased as the SE concentration increased. Luo et al. [26] investigated a preparation of W/O emulsions from tea polyphenol palmitate particles and found that the particle size of emulsified droplets decreased as the concentration of tea polyphenol palmitate particles increased. Figure 3b shows CLSM images of the oil phase stained with Nile Red, which is shown as red, and the aqueous phase is black. These images indicate that the emulsions prepared in the SE concentration gradient system were indeed W/O emulsions. The variation in the droplets was consistent with the results observed in the optical microscope.

**Figure 6:**
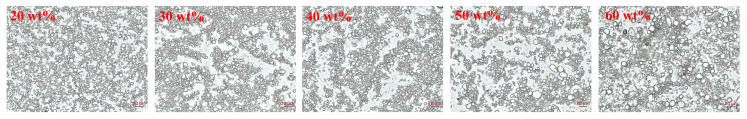
Microscopy images (scale bar = 20 μm) of fresh emulsions based on 1.5% SE and 0.5% BW complex systems with different aqueous phases.

After BW was introduced into the SE system, emulsions containing a 40 wt% water phase were successfully prepared under different ratios of SE and BW. Fresh emulsions with different mixing ratios all exhibited a uniform steady state. The prepared emulsions did not flow when inverted, and presented a gel state (Figure 1c). Figure 4 and Figure 5b show the size distribution and optical microscopy images of W/O emulsions with different SE and BW mixing ratios, respectively. It can be seen that the droplets stabilized by SE were irregular in shape and tended to form droplet clusters compared with those stabilized by BW alone, while the droplets stabilized by BW alone were mostly regular round droplets. In the 40 wt% aqueous phase condition, a small amount of BW was added to the SE emulsion system (1.5% SE: 0.5% BW), and the droplets became smaller. The addition of a small amount of BW made the droplets of the SE system more regular and homogeneous. This phenomenon might be due to the fact that a small amount of BW cooperates with SE to stabilize the interfacial film and enhance stability. However, when the amount of BW added continued to increase (i.e., 1% SE:1% BW and 0.5% SE: 1.5% BW), the droplets became larger and tended to aggregate. From the previous PLM image of the oil dispersions (Figure 2a), it can be seen that in canola oil, both BW and SE were spherical crystals [13,27]. As the extent of BW substitution for SE increases, BW and SE (both spherical crystals) can compete for adsorption to the interfacial membrane, which adversely affects the emulsion system originally stabilized by SE. Similar results were obtained by Khakhanang et al. [28], who prepared stable W/O emulsions using rice bran wax (RBX) and glycerol monostearate (GMS). They found that GMS altered the growth behavior of RBX when the wax content was relatively high, which further hindered the growth of wax crystals and reduced the emulsion stability. This was similar to the results of our microstructural diagram, which revealed a stable W/O emulsion at low BW content, where a small amount of BW acts synergically with SE to stabilize the emulsion. However, with the further replacement of SE by BW, BW and SE compete for adsorption at the oil–water interface at this time, so the optical and laser confocal microscope images reflect that the droplet size gradually increased. As shown in the PLM plots of Figure 4, the BW and SE crystals were dispersed in the continuous phase, and the dispersed phase was wrapped in the crystal network of the continuous phase. The figure also shows the crystal shell around the droplet (in the red box), which indicates that the crystals stabilize the emulsion at the interface. This result was similar to the findings of Hong et al. [18], who prepared W/O emulsions from candelilla wax and found that the wax crystal particles formed interfacial crystals at the interface, preventing droplet flocculation. In the CLSM images of Figure 4, the black spherical distribution in the oil phase can be clearly observed, indicating that the prepared emulsion was indeed a W/O emulsion. It can be seen that when a small amount of BW replaced SE, the droplet size of the W/O emulsion system decreased. When BW further replaced SE, the droplets were gradually larger. This result was consistent with the previous micrographs obtained with the optical microscope. Next, the ratio of SE to BW in the oil phase was fixed (1.5% SE: 0.5% BW), and the ratio of oil to water was adjusted to further study the stability of emulsions with higher water phases in this mixing system. As expected, with the increase in the internal water phase, it became more and more difficult to homogenize the emulsion, and it was impossible to prepare a complete W/O emulsion under 70% water phase. When the water content was less than or equal to 60%, the emulsions showed good stability and could be stored for one month without obvious phase separation (Figure 1d). Figure 6 shows the microstructure of W/O emulsions with different water phases, from which it can be seen that in the continuous phase of W/O emulsion with 20 wt% internal water phases, the regular droplets were relatively sparse. With the increase in internal water phase content, the connection between droplets became closer, and some droplets were found to be clustered together. This was due to the clustering of droplets resulting from the crystals of SE and BW acting as binders in the oil phase of the emulsion system [29]. It was also found that relatively irregularly shaped droplets appeared when the aqueous phase content was further increased above 50 wt%, and even some apparently deformed droplets as well as droplet coalescence were observed. Similar droplet aggregation phenomena were found in the studies of Khakhanang et al. [28] and Yu Lu et al. [30].

#### 3.2.2. Rheological Properties of W/O Emulsions

**Figure 7:**
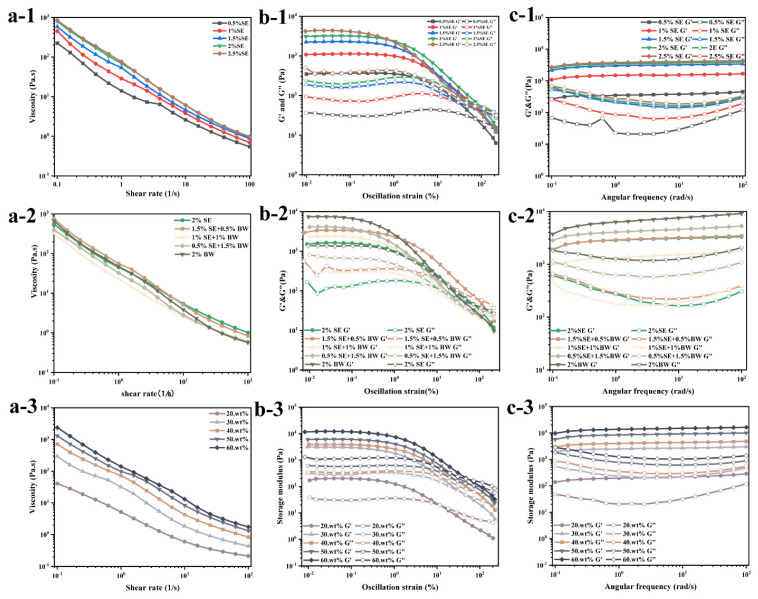
Shear rate sweep (**a-1**,**a-2**,**a-3**), strain sweep (**b-1**,**b-2**,**b-3**) and frequency sweep (**c-1**,**c-2**,**c-3**) tests of W/O emulsions from different systems.

Rheology plays an important role in evaluating the physical properties of emulsions, so it is considered an important parameter in clarifying the complex structure of emulsion products. A series of shear rate scanning, strain scanning and frequency scanning tests were performed on these different systems using the DHR-3 rheometer. Figure 7a shows three rheological scanning data graphs of water-in-oil emulsions with different SE concentrations. The apparent viscosity of the emulsions decreased exponentially with increasing shear rate and exhibited shear thinning behavior, indicating that the prepared emulsion systems were all yielding-pseudoplastic fluids (Figure 7a). In the concentration gradient system of SE, the concentration of SE increased, the particle size of droplets decreased, and droplets approached each other, forming an aggregated droplet and crystalline network, leading to an increase in the apparent viscosity of the emulsion system (Figure 7a-1) [13,26,31]. However, in the SE/BW ratio system, the system with the appropriate amount of BW added to SE showed the highest viscosity, and if BW continued to replace SE, it decreased the system viscosity. This might be due to the fact that in rapeseed oil, both BW and SE are spherical crystals, which can be more easily adsorbed at the interface due to their wettability as compared with other shapes of crystals [32]. Compared with the previous PLM images (Figure 2a), in the mixed system with the addition of appropriate amounts of BW and SE, both spherical and fine crystals were present, and the crystals were more likely to adsorb at the interface at this time than in other mixed systems, synergistically stabilizing the emulsion and enhancing its internal structure. At the same time, the viscosity of the system increased due to the interaction per unit volume [33]. When the proportion of BW continues to increase, the two fine crystals of SE and BW compete for adsorption at the oil–water interface, and the strength of the internal structure of the emulsion decreases so that it becomes susceptible to disruption and viscosity reduction [34]. Figure 7a-3 shows the flow scanning results of W/O emulsions (fixed SE and BW ratios) with different oil/water ratios. The results show that the viscosity increased with the increase in water content, which indicates that the increase in water content strengthened the network structure of the system.

The strain scanning was conducted at a fixed frequency of 1 Hz, and the scanning results of different systems are shown in Figure 7b. In all systems, all the samples showed the dominant behavior of “gel”, and in the whole LVR, G′ was a multiple of the corresponding G″. When the strain exceeded the critical value, the values of G′ and G″ decreased rapidly, crossed each other, and G″ was greater than G′, with the result that the internal structure of the system was damaged to some extent [35]. In Figure 7b-1, although the gel strength of the system was enhanced, the LVR width of the emulsion gradually decreased as the SE concentration increased. Among the systems with different SE and BW ratios, although the single BW system showed the highest elastic modulus, the LVR of this system was the narrowest, which indicates that the emulsion gel with single BW was more brittle than other emulsion gels, and the vibration yield stress (G′ = G″) was also the lowest [36]. However, when a moderate amount of BW was added to the SE system, the LVR expanded and the yield stress increased slightly compared with the single SE system, which further indicates that the moderate amount of BW and SE had some synergistic effect to improve the rheological properties of the emulsions. When the ratio of BW was further increased, the rheological properties of the prepared emulsions were inferior to those of the single SE system, which might be due to the fact that the excess BW competes with SE to adsorb at the oil–water interface, thus reducing the structural strength of the original SE system. The results of the amplitude scanning for different oil–water ratios (Figure 7b-3) show that the increase in the aqueous phase increased the structural strength of the emulsion and increased the LVR width. However, the yield stress was highest for the 40 wt% aqueous phases, while the yield stress started to decrease with continued increase in the aqueous phase. This might be due to the fact that although the increase in the water phase can improve the gel strength, too much water phase might promote the deformation of the droplets, leading to the easy destruction of their internal structure.

Figure 7c shows the results of the sweep frequency test of the emulsion sample. The results show that the G′ of W/O emulsions in all systems was greater than G″, which means that they all had properties similar to those of viscoelastic solids. The modulus of the emulsions had some frequency dependence in the different ratio systems of SE and BW, but the high frequency conditions had little effect on the rheological properties of the emulsions [36]. Combined with the previous flow scanning and strain scanning tests, it can be seen that the emulsion system stabilized by BW has a higher G′, but it has a narrower linear viscoelastic region, a lower yield stress than the single SE system, and a more brittle structure.

**Figure 8:**
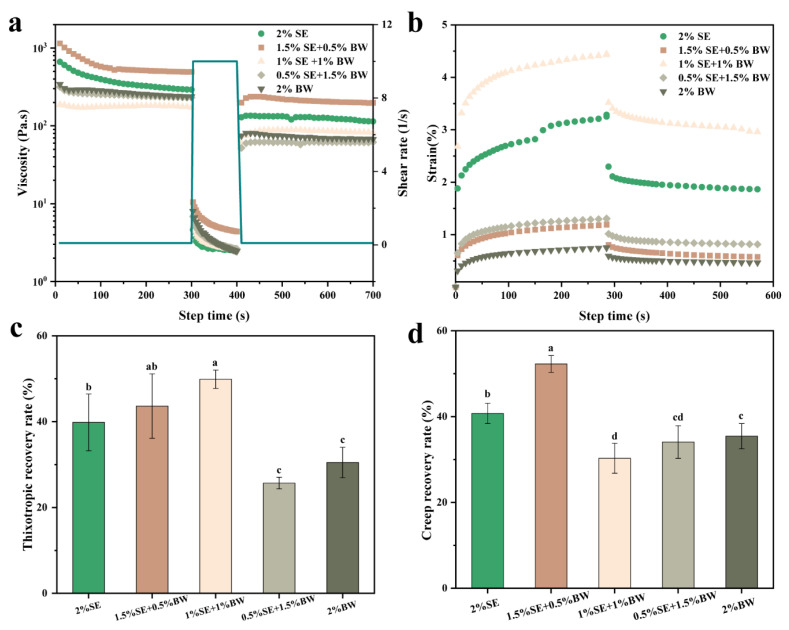
3ITT and creep-recovery experiments for W/O emulsions prepared with different SE and BW ratios (**a**,**b**), and their respective recoveries (**c**,**d**). The green line in (**a**) shows the shear rate applied in different time periods. Different letters in c and d indicate significant differences, *p* < 0.05.

Thixotropic and creep-recovery tests were carried out with emulsions with different SE and BW ratios. Figure 8a shows the scanning results of the thixotropy experiment, and explores the degree of emulsion structure recovery under different proportion systems. The figure shows the viscosity response of W/O emulsion gels under alternating low-speed and high-speed shear. The emulsions showed a time dependence, and the viscosity decreased with the extension of time at a fixed shear frequency. The moderate addition of BW improved the thixotropic recovery rate of the original SE system (Figure 8c). The creep-recovery test is a common method for evaluating the static viscoelasticity of emulsions. The effect of the ratio of SE to BW on the creep-recovery behavior of the emulsion system is shown in the Figure 8b. After constant stress was applied instantaneously, the samples of all the proportioning systems were deformed to varying degrees, and the strain increased with the extension of the action time. The creep-recovery response behavior of the samples reflects the strength of the internal structure of the emulsion samples in different ratios. The lower the strain response value, the higher the elastic behavior of resisting strain, and the stronger the structure of the emulsion [37]. The strain of the W/O emulsion prepared with a single 2% SE was only lower than that of the sample system prepared with 1% SE and 1% BW, indicating that the internal structure of the W/O emulsion sample prepared with a single SE was loose. W/O emulsions prepared with single 2% BW system showed the lowest strain response, indicating that W/O emulsions stabilized with BW had a relatively compact internal structure. When the W/O emulsion was prepared with the optimal BW/SE ratio, the strain response value was higher than that of the single BW emulsion system, and the creep-recovery rate was the highest (52.26%, Figure 8d), which indicates that the internal structure of the emulsion sample prepared with the original single SE was enhanced by an appropriate amount of BW and had better recovery ability.

**Figure 9:**
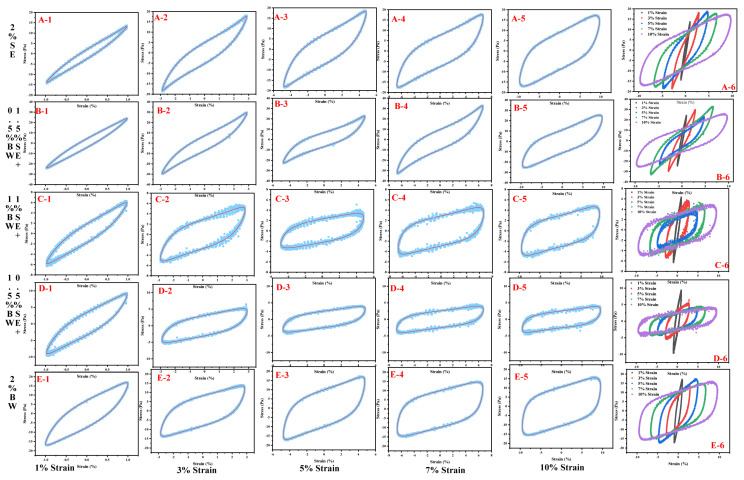
Elastic Lissajous–Bowditch diagrams of emulsions with a water content of 40% W/O prepared in different ratio systems of SE and BW.

In the nonlinear region with a lot of deformation, LAOS provides structural information as a quick qualitative graphic representation to better understand physical changes caused by deformation during processing or chewing [38]. Figure 9 is an elastic Lissajous–Bowditch diagram of emulsions with a water content of 40% W/O prepared with different proportions of SE and BW. Under the strain of 1%, the curve was oval regardless of the ratio of SE to BW, indicating that the sample was dominated by elastic behavior. With increasing strain, the shape of the curve was observed to be gradually distorted and deformed, indicating enhanced viscous dissipation, which was related to the permanent deformation and flow behavior of the microstructure of the emulsion [35]. Compared with the single SE system and the single BW system, with the increase in the proportion of BW in the SE system, the structure of the emulsion was greatly deformed under the strain, and the scanning results were relatively discrete, indicating that the emulsions formed with large proportions of BW and SE were loose and easily affected by the external force. After adding an optimal amount of BW to SE, the network structure and interactions between droplets were enhanced because both SE and BW formed spherical crystals and the droplets were tightly stacked to synergistically stabilize the emulsion.

## 4. Conclusions

In this study, W/O emulsion systems based on SE and BW were constructed, and it was found that appropriate amounts of BW and SE could synergistically stabilize W/O emulsions and improve their rheological properties. Under polarized light microscopy, both SE crystals and BW crystals were observed to be spherical. Further studies revealed that SE could not only form turbid oil-phase fluids, but could also form emulsion gels with viscoelasticity. Comprehensive observation of the microstructure diagrams of the emulsion droplets reveals that the droplets in all emulsion systems exhibited relatively obvious droplet aggregation, and the appropriate amount of BW helped stabilize the original SE-stabilized W/O emulsion system. This was mainly due to the presence of both spherical crystals and fine crystals in the 1.5% SE and 0.5% BW systems, which together stabilized the droplets and prevented small droplets from aggregating into large droplets. The results of rheological tests further indicated that appropriate amounts of BW could enhance the structural strength of the SE system, and the improved mechanical stability of the emulsions was indicated by high creep-recovery rates. In conclusion, this work provides a reference for further development of stable low-fat products.

## Data Availability

The datasets generated for this study are available on request to the corresponding author.
